# Demographic, Clinical, and Immunologic Features of 389 Children with Opsoclonus-Myoclonus Syndrome: A Cross-sectional Study

**DOI:** 10.3389/fneur.2017.00468

**Published:** 2017-09-11

**Authors:** Michael R. Pranzatelli, Elizabeth D. Tate, Nathan R. McGee

**Affiliations:** ^1^National Pediatric Myoclonus Center, Neuroimmunology Laboratory, Orlando, FL, United States; ^2^National Pediatric Neuroinflammation Organization, Inc., Orlando, FL, United States

**Keywords:** cerebrospinal fluid B cells, neuroblastic tumors, opsoclonus-myoclonus syndrome, neuroinflammation, paraneoplastic syndrome, pediatric neuroimmunological disorders, ROHHAD syndrome, anti-ANNA-1/anti-Hu syndrome

## Abstract

Pediatric-onset opsoclonus-myoclonus syndrome (OMS) is a devastating neuroinflammatory, often paraneoplastic, disorder. The objective was to characterize demographic, clinical, and immunologic aspects in the largest cohort reported to date. Cross-sectional data were collected on 389 children in an IRB-approved, observational study at the National Pediatric Myoclonus Center. Non-parametric statistical analysis was used. OMS manifested in major racial/ethnic groups, paralleling US population densities. Median onset age was 1.5 years (1.2–2 interquartile range), inclusive of infants (14%), toddlers (61%), and youngsters (25%). The higher female sex ratio of 1.2 was already evident in toddlers. Time to diagnosis was 1.2 months (0.7–3); to treatment, 1.4 months (0.4–4). Irritability/crying dominated prodromal symptomatology (60%); overt infections in <35%. Acute cerebellar ataxia was the most common misdiagnosis; staggering appeared earliest among 10 ranked neurological signs (*P* < 0.0001). Some untreated youngsters had no words (33%) or sentences (73%). Remote neuroblastic tumors were detected in 50%; resection was insufficient OMS treatment (58%). Age at tumor diagnosis related to tumor type (*P* = 0.004) and stage (*P* = 0.002). A novel observation was that paraneoplastic frequency varied with patient age—not a mere function of the frequency of neuroblastoma, which was lowest in the first 6 months of life, when that of neuroblastoma without OMS was highest. The cerebrospinal fluid (CSF) leukocyte count was minimally elevated in 14% (≤11/mm^3^) with normal differential, and commercially screened serum autoantibodies were negative, but CSF oligoclonal bands (OCB) and B cells frequency were positive (58 and 93%). Analysis of patients presenting on immunotherapy revealed a shift in physician treatment practice patterns from monotherapy toward multi-agent immunotherapy (*P* < 0.001); the number of agents/sequences varied. In sum, a major clinical challenge is to increase OMS recognition, prevent initial misdiagnosis, and shorten time to diagnosis/treatment. The index of suspicion for an underlying tumor must remain high despite symptoms of infection. The disparity in onset age of neuroblastoma frequency with that of neuroblastoma with OMS warrants further studies of potential host/tumor factors. OMS neuroinflammation is best diagnosed by CSF OCB and B cells, not by routine CSF or commercial antibody studies.

## Introduction

Opsoclonus-myoclonus syndrome (OMS) is a rare but serious neurologic disorder in children, with an estimated incidence of 0.18 cases per million of total population (1) or 0.27–0.40 cases per million children (2). It is associated with neuroblastoma—also rare (10.5 cases/million/year) (3) but the most common solid non-CNS tumor of childhood—and neuroinflammation, the instigator of a burgeoning group of serious neuroinflammatory disorders (4, 5). Stakes are high because the neurological and neuropsychiatric effects of OMS can be devastating and permanent (6–8), and the underlying cancer may be missed (9). The rarity of OMS, an orphan disease, has given rise to under-recognition, misdiagnosis, delay in treatment initiation, and even difficulty culling a sufficient population for research (9). Since 1962 when OMS (coined “myoclonic encephalopathy”) was first described in six children (10), no previous study has exceeded 105 cases (11–13), and most are small retrospective case series.

Neurologists, oncologists, and pediatricians alike are on the frontline when children present with OMS. The cardinal motor features include ataxia (imbalance, incoordination), myoclonus (body jerks originating from the CNS), or opsoclonus (random, multi-directional darting eyes movements) (4). Several OMS publications include patient videotapes (4, 14, 15). Signs may appear separately or together in a setting of non-motor features, such as irritability and insomnia (16). Later, neuropsychological sequellae, such as attention-deficit disorder, learning and memory disorders, and cognitive impairment, may emerge (6, 7). The challenge is to consider OMS early in the differential diagnosis (17), exclude look-alike diseases (4, 18–20), and shorten the time to treatment (12). Through such measures, the quality of life for children with OMS can be improved and the burden of chronic disease reduced. The present cross-sectional data were collected at a large specialty center for pediatric OMS with a goal of determining key demographic, clinical, and immunologic features of the disorder and helping to identify remaining barriers to early diagnosis and treatment.

## Patients and Methods

### Data Acquisition

#### Subject Recruitment

Data were collected beginning in 1989 through 2013 from 21 countries over five continents. Subjects with suspected or confirmed diagnosis of OMS were recruited by the National Pediatric Myoclonus Center, a specialty center for pediatric-onset OMS, and its website (www.omsusa.org). They were primarily domestic (357 from 49 states in the Mainland plus Alaska), but 31 were international (from 20 other countries, including Canada; Mexico; Czech Republic, Denmark, France, Germany, Greece, Hungary, Iceland, Italy, Poland, Portugal, Romania, Russia, UK; Bolivia, Chile; South Africa; Israel, and Saudi Arabia).

The National Pediatric Myoclonus Center moved sequentially from each of its former locations in the East (New York City, College of Physicians and Surgeons, Columbia University; Washington, DC, Children’s National Medical Center, The George Washington University) and Midwest (Springfield, IL, Southern Illinois University School of Medicine). It was incorporated as the National Pediatric Neuroinflammation Organization, Inc., an independent non-profit organization, in the State of Florida in 2015.

The first two authors moved with the center, providing patient care and research continuity and standardization of procedures. All patients traveled to the National Pediatric Myoclonus Center and were personally evaluated by Dr. Michael R. Pranzatelli, the principal investigator (clinician/scientist, pediatric neurologist) and Elizabeth D. Tate, the co-investigator (pediatric movement disorder-specialized nurse practitioner), who each have 30 years of pediatric OMS-specialty experience.

Socioeconomically disadvantaged US families who otherwise had no means to make the trip to our center were flown in by Miracle Flights for Kids (Green Valley, NV, USA) or Air Charity Network (formerly Angel Flight America). After 2001, they were boarded at Ronald McDonald House (Springfield, IL, USA) or local hotels at a discounted rate. This was done for compassionate reasons. Inclusion of low-income patients in the study prevented skewing toward those who could afford to fly.

This single center study was carried out in accordance with the recommendations of the respective IRBs (CUMC IRB, Children’s National IRB, and SCRIHS) with written informed consent from the parents (subjects were below age of consent). The parents of all subjects gave written consent in accordance with the Declaration of Helsinki. The protocol was approved by CUMC IRB, Children’s National IRB, and SCRIHS. Additionally, Western IRB designated IRB exemption for this retrospective analysis of demographic, clinical, and laboratory data.

A procedural summary is provided per 8-year study periods assigned retrospectively (Table 1). Briefly, out of 448 patients screened, 389 meeting inclusion/exclusion criteria were enrolled into this IRB-approved cross-sectional, observational study of patients at a single evaluation (not a longitudinal, interventional, or outcome study). Inclusion criteria comprised OMS diagnosis verified by the first two authors. Exclusion criteria included an incorrect diagnosis of OMS occasioned by findings of nystagmus or unrelated eye movement abnormalities, microcephaly with developmental delay, poor visual acuity, verified seizures (not just bouts of myoclonus), acute cerebellar ataxia, or other neurological disorder. Other exclusion criteria were skin conditions preventing lumbar puncture, severe anemia preventing blood drawing, and the presence of other autoimmune diseases. Patients were accepted at any stage in their course, including untreated and treated, which comprised those on treatment at the time and those no longer being treated. No treatments were given as a part of this study. Data about treatment refer only to what the patients were taking on arrival or had previously been given. All testing was performed at the National Pediatric Myoclonus Center.

**Table 1:** Summary of enrollment and clinical and laboratory evaluations per study period.

	Study period^a^			Start years	Specimen
	First	Second	Third		
Patient enrollment	+	+	+	1989	–
Clinical evaluations^b^	+	+	+	1989	–
Videotaping and scoring^c^	+	+	+	1989	–
**Laboratory testing**					
Routine cerebrospinal fluid (CSF) studies	+	+	+	1989	Fresh CSF
Paraneoplastic antibodies		+	+	1997	Frozen serum
Flow cytometry		+	+	2001	Fresh CSF and blood
Oligoclonal bands		+	+	2002	Frozen CSF and serum

*^a^The three study periods were 1989–1997, 1997–2005, and 2005–2013*.

*^b^History and videotaped neurological examination performed by child neurologist and child neurology-specialized nurse practitioner*.

*^c^The OMS video evaluation scale was published in 2001 (21)*.

#### Clinical Data

The investigators took medical histories, collecting data on all clinical symptoms of OMS, and performed neurological examinations. All examinations were performed by the same examiners for standardization and reliability.

Motor severity was semi-quantitated using a Likert-like video evaluation scale (15). Parents gave written consent for videotaping, and the videotapes were used to score OMS motor severity based on a 12-item scale, a standard of care described and validated previously (21). To maintain rater blinding, videotapes were cued up by an assistant and scoring was done in batches. Ratings of 0–3 were given for each item according to written criteria (21) and added to create a “Total Score” of 0–36.

Parents were asked to number the order of appearance of neurological signs in OMS from a written list of 10 lay-oriented, characteristic signs using the following terms: body jerks, drooling, falling, irritability, limpness, loss of speech, opsoclonus, refusing to walk, staggering, and unable to sit.

#### Laboratory Data

All CSF and serum samples were collected at the National Pediatric Myoclonus Center. Various measures were taken to standardize laboratory procedures and protect the quality of the data.

Cerebrospinal fluid leukocyte count with differential, protein, and glucose were quantitated in the clinical lab. CSF cells were counted manually with a hemocytometer.

Oligoclonal bands (OCB)—those found in CSF but not in matching serum—were measured by isoelectric focusing with immunofixation (22). Only OCB testing by isoelectric focusing with immunofixation, the preferred method, was done. It was performed almost exclusively through ARUP Lab (Salt Lake City, UT, USA) after 2004, having been done at Mayo Clinic Lab (Rochester, MN, USA). ARUP reports the exact number of bands and designates two bands (rather than four) as positive.

The frequency of CSF B cells (CD19^+^CD3^−^) was determined in the clinical flow cytometry lab as previously described (15). Flow cytometric analysis of CSF lymphocytes requires collection on ice and processing within 1 h; the sample cannot be mailed. Blood leukocytes are viable for longer, but were processed as promptly.

Serum was screened commercially for paraneoplastic autoantibodies. Early commercial paraneoplastic antibody screening of serum was done by enzyme-linked immunosorbent assay and Western blot analysis. Before 1996, Specialty Laboratories, Inc. (Santa Monica, CA, USA) provided screening for anti-Hu and anti-Yo. Then Athena Diagnostics (Worchester, MA, USA) provided an anti-Hu, anti-Ri, and anti-Yo panel. After 2004, Mayo Clinic Lab screened for an expanding group of multiple putative autoantibodies. During that period, the antibody terminology changed to immunohistochemical designations: anti-Hu became ANNA-1; anti-Ri, ANNA-2; anti-Yo, PCA-1. Also, screening technology changed to using cell-based assays, with Western blots only to verify positives. Paraneoplastic antibodies can be measured in CSF, but serum titers are usually higher, and CSF testing is usually reserved for positive sera.

Our published control B cell and OCB data were obtained from children undergoing lumbar puncture to rule out neuroinflammation but who were found to have non-inflammatory neurological disorders. These included headache, ataxia, developmental delay, movement disorders, seizure disorders, and miscellaneous disorders (15, 22). For interpretation of commercial autoantibody tests, the laboratory’s own reference ranges were used.

### Statistical Analysis

Descriptive statistics and graphics were prepared by the third author using GraphPad Prism (San Diego, CA, USA), consulting with statisticians as needed. SAS (Cary, NC, USA) was used for statistical tests not available in GraphPad. The analysis included the 105 cases in reference (11), which was a questionnaire study and did not involve neuroimmunological testing. Chi square or Fishers Exact test was used to compare frequencies. Medians were compared using Mann–Whitney or Kruskal–Wallis (K–W) tests, with the Dunn *post hoc* test for the latter. The interquartile range (IQR) was used as a measure of statistical dispersion and calculated as the difference between the upper (75th percentile) and lower (25th percentile) quartiles. A Gaussian curve was fitted to the amount of time between onset of OMS symptoms and diagnosis. Data on the parent-numbered order of presenting neurological signs were ranked, because one number is dependent on the next, not all signs are present in every patient, and the number of parents responding varied. A *P*-value of <0.05 was considered statistically significant throughout. Data were stratified for analysis by age of OMS onset, OMS treatment status (untreated vs treated), and tumor status. The focus was on untreated patients because of their comparative rarity and the absence of obscuring treatment effects. Bonferroni corrections were used for multiple statistical comparisons. Missing data were categorized as “missing at random.” The pattern of “missingness” was arbitrary and likely to produce little or no bias or distortion in the conclusions.

The long duration of the study also afforded an opportunity to retrospectively evaluate changes in clinical practice patterns and in tumor detection over the years. In secondary statistical analysis, it was divided into three equal periods. In other secondary analysis, the main demographic and clinical data from the group of international patients were analyzed separately to determine if they were homogeneous with the overall dataset.

## Results

### Demographic Characteristics

#### Geographical Distribution

**Figure 1:**
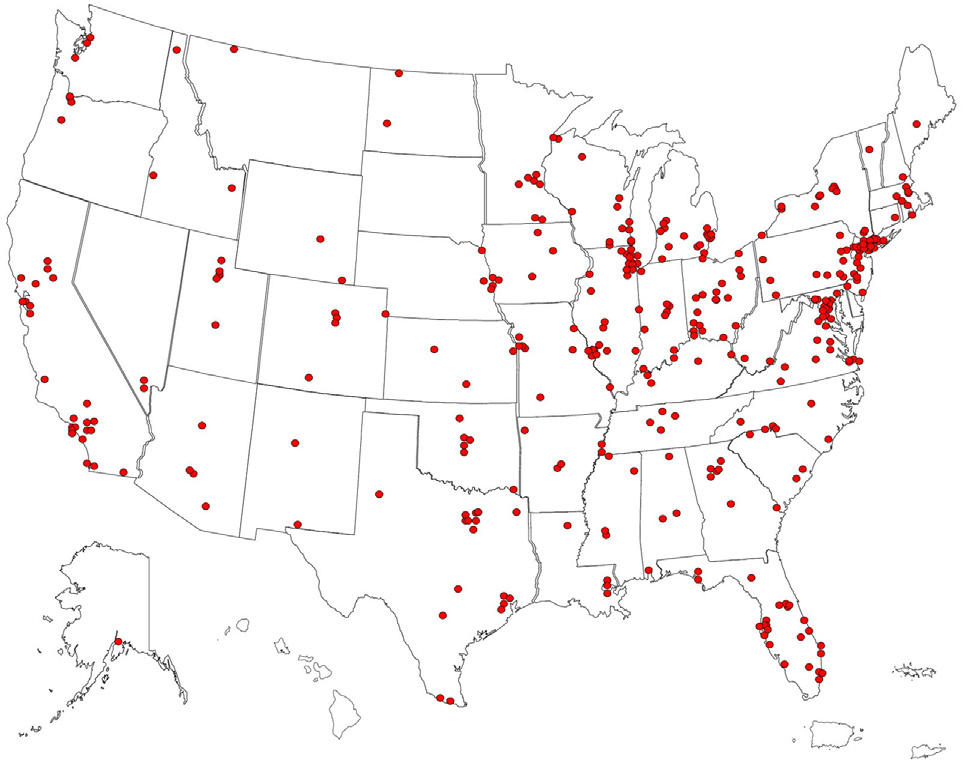
US pin map of residential locations of children with opsoclonus-myoclonus syndrome (OMS) in the study sample. Some dots overlie each other. Our former center locations drew from the Mid-Atlantic States and Midwest. Fewer cases came from Southern California, where there is another OMS center.

Based on data about the geographical distribution of the US patient sample (*N* = 358) (Figure 1), OMS densities corresponded to US population densities, with the most densely populated parts lying east of the Mississippi River. There were major OMS visual aggregates around highly populated New York City, Washington, DC, and Chicago; smaller clusters in Seattle, San Francisco, Los Angeles, St. Louis, Tampa/St. Petersburg, and New Orleans. Although both urban and rural areas are represented, most cases were in or around cities and suburbs, as for 81% of the US population (23).

#### Race, Ethnicity, and Gender

Opsoclonus-myoclonus syndrome occurred in the major racial and ethnic groups, which were self-identified (Table 2). The distribution of US patients corresponds well with the current US Census (14). Though statistical comparison was infeasible, no apparent racial or ethnic predilection for OMS was observed. There were 10% more females than males.

**Table 2:** Demographics of opsoclonus-myoclonus syndrome (OMS).

Category	All OMS (*N* = 389)	US OMS (*N* = 358)	US Census^a^ (322 million) (%)
Age, years (median, interquartile range)	2.7 (1.9–4.0)		
**Gender**			
Male (*n*, %)	174 (45%)	158 (44%)	
Female (*n*, %)	215 (55%)	200 (56%)	
**Race/ethnic groups**			
White, non-Hispanic	274 (70%)	252 (70%)	64
Hispanic/Latino	58 (15%)	57 (16%)	16.4
Black	38 (10%)	32 (10%)	12
Asian/Oceanic	14 (4%)	12 (3%)	4.7
American Indian	5 (1%)	5 (1%)	0.7

*^a^US Census Bureau (23)*.

### Prodrome

**Figure 2:**
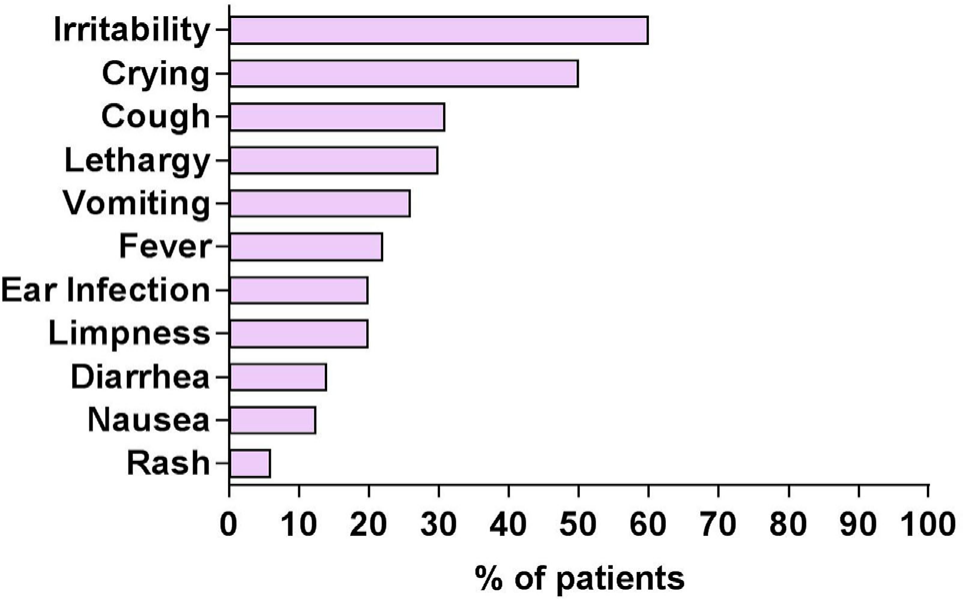
Prodromal symptoms in patients with opsoclonus-myoclonus syndrome (OMS) per history. Patients may have more than one prodromal symptom.

The parent-reported frequencies of 11 prodromal symptoms of OMS were analyzed (Figure 2). Sixty percent of patients displayed irritability (usually with crying) prior to OMS onset. Twenty-two percent of total patients were said to be febrile. Symptoms suggestive of a possible non-specific upper respiratory illness (cough/ear infection) occurred in 31%/20%; a possible gastrointestinal illness (vomiting/diarrhea) in 26%/14%. Not all patients manifest every prodromal symptom. In 40%, no prodromal symptoms were noted. Patients had been tested for various infectious organisms, and many underwent a lumbar puncture, though the comprehensiveness of outside CSF studies varied greatly. No consistent type of infection was documented by clinical or laboratory measures.

### OMS Characteristics

#### Age-of-Onset and Duration

**Figure 3:**
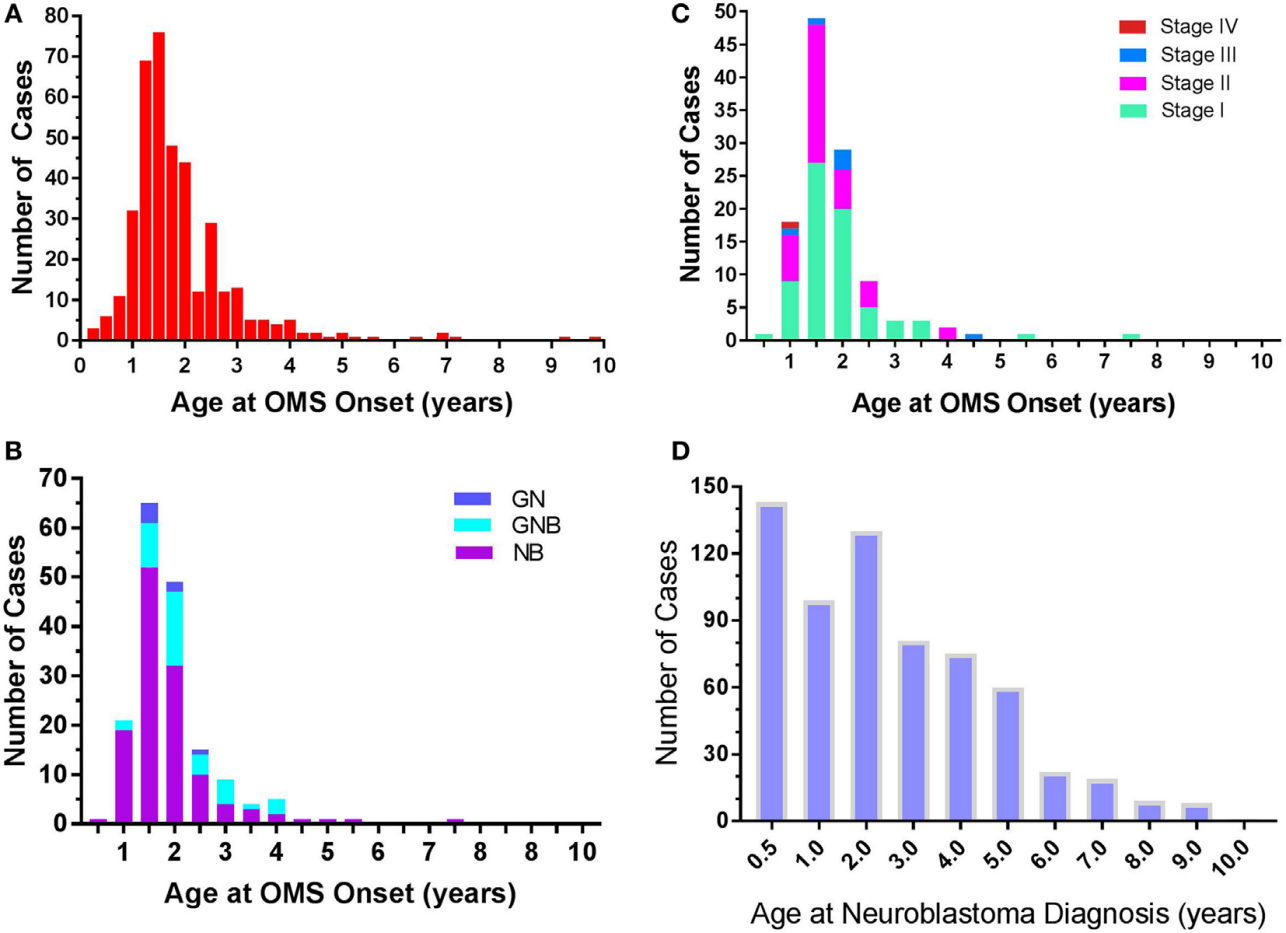
**(A)** Frequency of opsoclonus-myoclonus syndrome (OMS) by age of OMS onset. *N* = 389. Data are displayed at 0.25-year increments. **(B)** Frequency of various neuroblastic tumors by OMS onset age. *N* = 177. Abbreviations—NB, neuroblastoma; GNB, ganglioneuroblastoma; GN, ganglioneuroma. **(C)** Frequency of neuroblastoma INSS stages by OMS onset age. *N* = 118. Classification—Stage 1: localized unilateral tumor, negative lymph nodes outside tumor; Stage 2: not all visible tumor could be resected but negative lymph nodes outside tumor (2A) or positive ipsilateral nodes (2B); Stage 3: not completely resected, tumor crossed midline, or positive contralateral lymph nodes, or tumor midline and spread to both sides; Stage 4: metastatic; Stage 4S: metastatic in infants <1 years old, unilateral tumor, no contralateral positive nodes (<10% marrow cells positive). **(D)** Frequency of neuroblastoma without OMS from published statistics (3). *N* = 643. Visual comparison of **(C,D)** reveals that the frequency of paraneoplastic OMS and of neuroblastoma is not a mere function of each other.

Among all 389 cases, the reported onset of OMS symptoms was most common in toddlers (Figure 3A), but ranged from 0.17 to 9.8 years. A best-fit Gaussian curve placed the peak at 1.5 years (0.47), with most patients accounted for between 0.5 and 3 years, and the amplitude at 69.5 (number of patients). OMS onset in infants ≤ 6 months of age occurred in only 2% of all OMS. OMS duration was acute/subacute in 57% of the patients.

#### Neurological Signs

In untreated OMS (*n* = 74), clinical signs were heterogeneous. Opsoclonus, myoclonus, ataxia, and insomnia were not exhibited by 100% of patients at our initial evaluation (Table 3). Opsoclonus showed inter- and intra-individual variation in intensity and frequency, ranging from rare to florid. Thirteen percent of youngsters exhibited ocular flutter instead. Myoclonus gave rise to a tremulous appearance that could be confused with tremor in mild cases, but became characteristically jerk-like with increasing severity and was action-induced. The median Total Scores fell into the moderate severity range.

**Table 3:** Clinical features and treatment history of opsoclonus-myoclonus syndrome (OMS) (*N* = 389).

Feature	Data			
	Median (IQR)		*N*	%
Age of OMS onset, years	1.5 (1.2–2.0)			
OMS duration, years	0.8 (0.3–1.9)			
Time to OMS diagnosis, months	1.2 (0.7–3.0)			
Time to treatment, years	1.4 (0.4–4.0)			
Length of follow-up, years	1.9 (1.0–3.4)			
**“Total Score” (OMS severity)^a^**	15 (9–22)			
Untreated OMS	21 (15–25)		74	19
Arriving on treatment	14 (9–23)		233	60
Treated previously only	14 (8–20)		82	21
**OMS duration category**				
Acute (≤3 months)			97	25
Subacute (>3 to <12 months)			125	32
Chronic (≥12 months)			167	43
**In untreated OMS, features (history/intake)^b^**				
Opsoclonus			61	82
Myoclonus			66	89
Ataxia			55	75
Insomnia			53	72
**In treated OMS, categories^c^**				
Monotherapy			114	49
Dual therapy			86	37
Triple therapy			25	11
Other multiple agents			8	3
**Treatment agents (*N* = 233)**				
Corticosteroids only			35	15
Corticotropin only			43	18
IVIg only			34	15
Steroids and IVIg only			40	17
Corticotropin and IVIg only			46	20
Agents in additional combinations			35	15
Cyclophosphamide	20	57		
Rituximab	10	28		
Azathioprine	2	6		
Mycophenolate mofetil	3	9		

*IQR, interquartile range*.

*^a^Total Score designates mild (0–12), moderate (13–24), and severe (25–36) cases*.

*^b^Percentages do not tally to 100 because the features are not mutually exclusive*.

*^c^“Monotherapy” and “Dual Therapy” refer to corticosteroids, corticotropin, or intravenous immunoglobulin (IVIg). Triple Therapy, mostly front-end multimodal, also includes rituximab or low-dose cyclophosphamide. “Other Multiple Agents” also includes steroid sparers*.

*The inset N and % values for agents in additional combinations were tallied separately*.

**Figure 4:**
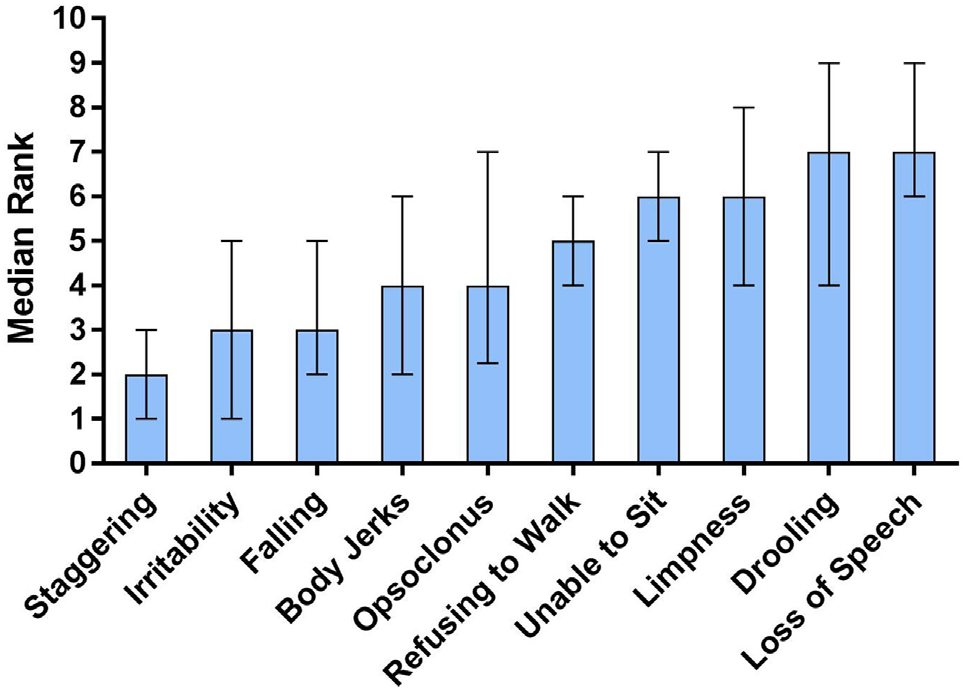
Ten presenting neurological signs of opsoclonus-myoclonus syndrome (OMS) by order of appearance per parents. The data are median ranks with interquartile range: the lower the rank, the earlier the sign. In Dunn’s multiple comparisons test, staggering occurred significantly before other signs (*P* < 0.0001). Refusing to walk and inability to sit were similar in occurring significantly later than falling, body jerks, and irritability, but earlier than loss of speech. Falling and body jerks occurred earlier than loss of speech or drooling. Loss of speech and drooling occurred later than irritability.

In the ranking by parents of 10 presenting neurological signs of OMS (Figure 4), staggering occurred before all other signs (*P* < 0.0001). The median age-of-onset for each of the 10 neurological signs also was compared statistically (*N* = 339). It was significantly lower for opsoclonus (1.25 year, 0.94–1.57) than for ataxia (1.67 year, 1.36–2.47) and irritability (1.58 year, 1.17–2.42) *(P* = 0.0007).

The presentation of neurological abnormalities differed by age-of-onset of OMS (Table 4). In untreated patients with complete data, 20% of toddlers and 22% of youngsters exhibited choking or trouble swallowing liquids acutely. There were no fatalities. Lack of vocabulary characterized 89% of toddlers and 33% of youngsters. One-hundred percent of toddlers and 73% of youngsters could not form sentences, which also was assessed as delayed.

**Table 4:** Stratification of clinical and laboratory data by opsoclonus-myoclonus syndrome (OMS) onset age: three subgroups.

Feature	OMS age-of-onset (months)			*P*-value*^a^*
	Infants ≤12	Toddlers 12–24	Youngsters >24	
**Clinical features in all OMS (including treated)**				
*N*	53 (14%)	240 (61%)	96 (25%)	–
Within group median onset age, years	0.83	1.5	2.8	–
Sex ratio (F:M)	1.0	1.29	1.23	NS
Tumor found, Y:N	31:22 (58%)	126:114 (52%)	37:59 (38%)	0.03
Type (NB:GNB:GN)^b^	25:4:0	87:24:6	22:14:1	0.04
**Laboratory abnormalities in untreated OMS**				
*N*		40	26	–
CSF tests, median (IQR)^c^				
WBC count (/mm^3^)		1.5 (0–3.2)	3 (0–4.5)	NS
OCB count		1.5 (0–8.2)	4 (1.5–6.5)	NS
% OCB positive		54	83	–
CSF B cell percentage		3.8 (2.2–7.3)	3.9 (3.2–7.1)	NS
**Neurological abnormalities in subset of untreated OMS^d^**				
*N*		18	15	
History				NS
Choking/liquids		4 (22%)	3 (20%)	
Trouble with speech		10 (56%)	11 (73%)	
Drooling		12 (67%)	11 (73%)	
Irritability		9 (50%)	10 (67%)	
Disturbed sleep		11 (61%)	12 (80%)	
Neurological exam				NS
Opsoclonus		11 (61%)	10 (67%)	
Ocular flutter		0	2 (13%)	
Action myoclonus		9 (50%)	9 (60%)	
Arm dysmetria		5 (28%)	5 (33%)	
Gait ataxia		10 (56%)	11 (73%)	
Dysarthria		3 (17%)	11 (73%)	
Absence of words		16 (89%)	5 (33%)	
No sentences		18 (100%)	11 (73%)	

*NB, neuroblastoma; GNB, ganglioneuroblastoma; GN, ganglioneuroma; IQR, interquartile range; CSF, Cerebrospinal fluid; OCB, oligoclonal bands; WBC count, leukocytes*.

*^a^Fishers exact tests. Not statistically significant after Bonferroni corrections requiring P < 0.017*.

*^b^Neuroblastoma was commonest at any age-of-onset, but the proportion of tumor types changed with later onset*.

*^c^CSF OCB count ≥2 is designated as elevated by reference lab. CSF B cell frequency >1% is above the control median, and ≥2% is designated as abnormal*.

*^d^Comprises 33 untreated OMS with complete data. For infants, there were insufficient data for statistical analysis*.

The rest of the neurological examination was within normal limits. Head circumference was age-appropriate. Cranial nerves were intact (II–XII in older children). Deep tendon reflexes in the lower extremity (*N* = 137) were 0–1+ in 32%; 2+ in 45%; and 3+ in 23%. Twelve percent had clonus, which was not sustained. In testable children, light touch and proprioception were normal. Muscle tone was decreased to normal; strength was intact. There were no other adventitious movements.

### Tumor Characteristics

#### Type, Stage, Location, and Frequency

In the 50% of patients in whom a tumor was found (*n* = 194), the tumor was neuroblastic: neuroblastoma in 73%, particularly with younger OMS onset age (Table 5). The proportion of ganglioneuroblastoma increased with onset age but neuroblastoma predominated (Figure 3B). In infantile-onset OMS, neuroblastoma occurrence was 6.2-fold greater than ganglioneuroblastoma; 3.6-fold in toddler-onset OMS; and 1.6-fold in youngster-onset OMS. In 90% of cases of OMS with a tumor, the tumor was diagnosed by 1.6 years of age (1.33–2.05; *N* = 119).

**Table 5:** Overall tumor information.

Parameter/variable	*N*	%	Median (interquartile range)	*P*-value
**Tumor detection^a^**				
Entire study (*N* = 389)	194	50		
First study period (*N* = 41)	16	39		
Second study period (*N* = 92)	48	52		
Third study period (*N* = 256)	130	51		
**Location (*N* = 178)**				
Adrenal	48	27		
Abdominal/paraspinal	65	36		
Thoracic/paraspinal	50	28		
Pelvic	11	6		
Cervical	4	2		
Not available	16	–		
**Type (*N* = 183)**				
Neuroblastoma	134	73		
Ganglioneuroblastoma	41	22		
Ganglioneuroma	8	4		
Not available	11	–		
**INSS neuroblastoma stage (*N* = 118)**				
I	68	58		
II	42	35		
III	6	5		
IV	2	2		
Not available	16	–		
**Chemotherapy for tumor (*N* = 150)**				
Yes^b^	20	13		
No	130	87		
**Effect of tumor resection on OMS (*N* = 134)**				
Improved	56	42		
Unchanged	50	37		
Worsened	28	21		
Age (years) at tumor diagnosis vs tumor type				0.004*^c^
Neuroblastoma			1.6 (1.2–2.0)	
Other neuroblastic tumors			1.9 (1.5–2.9)	
Age (years) at neuroblastoma diagnosis vs stage				0.002*^c^
I			1.5 (1.0–1.7)	
II			1.7 (1.5–2.4)	
Effect of age (years) at tumor diagnosis vs effect of tumor resection on OMS				
				0.03^c^
No change/better			1.7 (1.4–2.3)	
Worse			1.5 (0.9–2.0)	

*INSS, International neuroblastoma staging system; OMS, opsoclonus-myoclonus syndrome*.

*^a^All tumors were surgically resected*.

*^b^Includes only patients treated with chemotherapeutic agents for their tumor (adriamycin, cisplatin, cyclophosphamide, dacarbazine, VP16), not for OMS*.

*^c^Mann–Whitney tests*.

*Statistically significant after Bonferroni corrections requiring P < 0.017.

INSS Stage I neuroblastoma predominated, especially in children with earlier OMS onset. Stage I and II accounted for 93% of neuroblastoma with documented staging, whereas Stages III and IV occurred infrequently (7%). In the whole dataset, an adrenal or abdominal/paraspinal location was most common (63%), followed by thoracic/paraspinal (28%); pelvic and cervical locations were uncommon but noteworthy (8%).

Comparing the frequency of neuroblastoma with OMS (Figure 3C) to that of neuroblastoma without OMS (Figure 3D), the frequency of paraneoplastic OMS was not a simple function of neuroblastoma frequency. In contrast to the toddler years, paraneoplastic OMS was under-represented in infants paradoxically, and its frequency fell more steeply thereafter than did neuroblastic tumor frequency.

#### Tumor Detection by Study Period

Tumor detection in the second and third study periods was comparably higher (51–52%) than in the first study period (39%) (*P* = 0.02, Chi square). The neuroblastoma diagnostic evaluation was variable, ranging from only chest X-ray and abdominal ultrasound to body cavity CT or MRI and/or metaiodo-benzyl-guanidine (MIBG) scans. Screening tended to be more comprehensive in the past decade. There were two false positive MIBG scans, leading to unnecessary surgery, and five false negative MRI scans, not confined to one study period.

#### Tumor Resection

Tumor resection prior to the evaluation had been associated with improvement (though usually insufficient) in 42%, but no change or worsening of OMS in 58%. Worsening was slightly more common in younger children. Permanent post-surgical neurological complications were uncommon, but included Horner’s syndrome (several cases), sympathetic dystrophy of one leg (one case), and scoliosis (from spinal foraminal encroachment). Some patients had received chemotherapy to shrink the tumor to a resectable size or because of higher tumor stage, but their subgroup was too small for statistical analysis.

### Neuroinflammation

Minimal CSF pleocytosis (CSF WBC > 4/mm^3^) was found in 14% of untreated OMS, all ≤11/mm^3^. The median WBC differential was 78% lymphocytes (IQR, 64,87), 21% monocytes (13,34), 0% neutrophils (0,1), and 0% segmented cells (0,0.75). One patient had two bands; none had eosinophils or basophils. CSF glucose and protein were also normal (data not shown). There was no significant effect of OMS onset age on the total CSF leukocyte count in OMS, based on results in toddlers and youngsters (Table 4). The infant OMS group remains an interesting potential comparison group, but there were too few data to report.

B cell frequency was elevated in CSF, not blood, as previously demonstrated. What is new is the comparison between CSF B cell percentages and the age of OMS onset. No statistically significant difference in the extent of CSF B cell expansion was found between toddlers and youngsters. Both groups mounted a significant intrathecal B-cell response.

Cerebrospinal fluid OCB counts were significantly elevated compared to case-controls, and there was a spread in range, but they did not differ significantly between toddlers and youngsters. However, only the OCB median in youngsters was above the laboratory threshold for positive (≥2). The frequency of patients with positive OCB appeared higher in youngsters (83%) than toddlers (54%).

Commercially screened serum IgG autoantibodies were negative except for subclinical titers of one of two antibodies in four patients: 3 with anti-GAD/anti-GAD65 and 1 with anti-neuronal V-G potassium channel. None of the following paraneoplastic antibodies were found: anti-neuronal nuclear (types-1, 2, 3), anti-glial nuclear (type 1), Purkinje cell cytoplasmic (types 1, 2, and Tr), amphiphysin, NMDAR, AMPAR, GABA-B, CRMP-5, or calcium channel (N1 or P/Q-type). In contrast, evidence of neuroinflammation and immune dysregulation was demonstrated in untreated OMS by positive CSF OCB (58% of patients) and the pathologically increased frequency of CSF B cells (93% of patients). In patients arriving on treatment, OCB positivity was 27%.

### Treatment Status at Initial Visit

#### Immunotherapy Types

Of patients who arrived on immunotherapy (*n* = 233) (Table 3), 86% were on one or two agents; 14% were on multiple agents, either as directed multimodal therapy or as a result of adding agents over time based on response. Monotherapies included corticosteroids, corticotropin, or intravenous immunoglobulin (IVIg). Two-agent therapies included either corticosteroids or corticotropin and IVIg. The third agents comprised cyclophosphamide, rituximab, or steroid sparers (azathioprine, mycophenolate mofetil).

#### Changes in Practice Patterns

Opsoclonus-myoclonus syndrome treatment practice patterns were compared by study period (Table 6). Period 1 accounted for 8% of the number of cases during the study periods; period 2, 24%; and period 3, 68%. The main difference between the first and third study periods was the use of rituximab and of combination immunotherapy in the latter. The second and third periods, which resembled each other (except for the use of rituximab and disuse of azathioprine and mycophenolate in the third study period), stood out from the first study period due to use of three or more agents or of steroid sparers. The treatment categories in the combined second and third periods vs the first period were significantly different (*P* < 0.0001).

**Table 6:** Immunotherapy practice patterns.

	Study period				*P*-value
	First (1989–1997)	Second (1997–2005)	Third (2005–2013)	Second + third	
*N* 233^a^	19 (8%)	56 (24%)	158 (68%)	214	–
Treatment categories^b^					<0.001*^c^
One agent	13 (68%)	27 (48%)	74 (47%)	101 (47%)	
Two-agents	6 (32%)	23 (41%)	65 (41%)	88 (41%)	
Three or more agents	0	6 (11%)	19 (12%)	25 (12%)	
Treatment agents					<0.001*
Monotherapies					0.02
Corticosteroids only	2 (10%)	9 (16%)	24 (15%)	33 (15%)	
Corticotropin only	7 (37%)	11 (20%)	25 (15%)	36 (17%)	
IVIg only	4 (21%)	6 (11%)	24 (15%)	30 (14%)	
Combination therapies					<0.001*
Steroids and IVIg only	2 (10%)	7 (12%)	31 (20%)	38 (18%)	
Corticotropin and IVIg only	4 (21%)	12 (21%)	30 (19%)	42 (20%)	
Multiple agents	0	11 (20%)	24 (15%)	35 (16%)	
Types of multiple agents					n.a.
Cyclophosphamide	0	6 (55%)	14 (58%)	20 (57%)	
Rituximab	0	0	10 (42%)	10 (28%)	
Azathioprine	0	2 (18%)	0	2 (6%)	
Mycophenolate	0	3 (27%)	0	3 (9%)	

*IVIg, intravenous immunoglobulin*.

*^a^Patients on active treatment at time of initial evaluation by the NPMC*.

*^b^N and % by column*.

*^c^Chi square test of first period vs combined second and third period. Individual treatment periods did not differ statistically, but the combined second and third periods, which were alike, differed significantly from the first period*.

*Statistically significant after Bonferroni corrections requiring P < 0.012.

*n.a., individual comparisons of other agents in combination therapy could not be performed for rituximab due to 0 values for first and second study periods*.

### Secondary Analysis of International Patients

As to group demography, 55% were European; 19% Canadian; 10% Middle Eastern; 6.5% Mexican; 6.5% South American, and 3% South African. The race/ethnicity breakdown was 71% White, non-Hispanic; 19% Hispanic; 3% Black, and 7% Asian. There were 52% males (*n* = 16) and 48% females (*n* = 15).

The clinical and neuroimmunological features of the international patients were more similar than different compared to those in the entire OMS dataset (Table 7). There were no notable group differences in the OCB and CSF B cell profile. OMS onset age was identical; median patient age was 7 months older. The international patients tended to be more chronic by 25%, less acute; had an 18% higher tumor frequency; and tumor type was slightly shifted from neuroblastoma (down 14%) toward other neuroblastic tumor types. The higher percentage of treatment with three or more immunotherapeutic agents and fewer monotherapies in the international group may be an artifact of a higher proportion of patients in study period 3. Due to the discrepant sizes of the two groups, no further analysis was conducted.

**Table 7:** Secondary analysis of opsoclonus-myoclonus syndrome (OMS): international group (*N* = 31).

Feature	Data		
	Median (interquartile range)	*N*	%
Patient age, years	3.3 (2.2–4.7)		
Age of OMS onset, years	1.5 (1–1.8)		
OMS duration, years	1.4 (0.62–3.4)		
Total score (OMS severity) (*N* = 29)	16 (11–25)		
Untreated	(13, 20)	2	7
Arriving on treatment	15 (10–24)	19	65
Treated previously only	16 (10–26)	8	28
**OMS severity category**			
Mild (TS 0–12)		8	28
Moderate (TS 12–24)		12	41
Severe (TS 25–36)		9	31
**OMS duration category**			
Acute (≤3 months)		2	6
Subacute (>3 to <12 months)		8	26
Chronic (≥12 months)		21	68
**Tumor**			
Yes		21	68
No		10	32
**Tumor type**			
NB		10	59
GNB		5	29
GN		2	12
Not available		4	–
**CSF OCB (N = 25)**			
Positive (≥2)		14	56
Negative (<2)		11	44
**CSF % B cells (N = 27)**			
Positive (≥2%)		21	78
Negative (<2%)		6	22
**Treatment category (N = 29)**			
1 agent		5	17
2 agents		12	41
3 agents		7	24
≥3 agents		5	17
**Treatment agents**			
Corticosteroids		22	
Prednisone	3		
Prednisolone	2		
Methylprednisolone	1		
Dexamethasone	5		
Deflazacort	1		
Corticotropin (1–39)		11	
Corticotropin (1–24)		4	
Intravenous immunoglobulin		22	
CPM		5	
RTX		3	
Chemo		9	
Azathioprine		2	
**Study period**			
First		1	3
Second		5	16
Third		25	81

*There were only two untreated patients, no median value*.

*Of patients treated previously only: 5 had received a single agent; 22, more than one agent; 4, untreated. Of patients arriving on treatment: 10 on single agent, 10 on more than one agent*.

*Deflazacort: 6 mg is equivalent to 5 mg of prednisolone*.

## Discussion

This report reflects the largest pediatric-onset OMS cohort to date. For the first time, it was of sufficient size to make critical subgroup comparisons. The study adds new demographic, clinical, and laboratory observations and extends findings from our questionnaire-based early report (11). For clarity, the key findings are discussed under their respective subheadings and stated as topic sentences of each paragraph.

### Demographic

The main demographic observation was that more females than males had OMS, and the slight female preponderance was first detected in toddlers and consistent thereafter. In the present study, 55% of patients were female. In the UK (12), a study of 101 cases found 52% were female; of 23 cases in Japan (2), 56% were female; in France (24), 65% of 34 cases were female; in a study of 32 cases from multiple countries, including the US, 62.5% were female (25). Adding our 389 cases to those 190 cases (*N* = 579), 322 were female (56%) and 257 (44%) were male. This analysis documents a small consistent female preponderance in OMS in multiple countries. Risk factors for female children with neuroinflammatory/autoimmune disorders merit further study.

### Neurologic

The earlier appearance of ataxia than opsoclonus or myoclonus in OMS may provide one reason why acute cerebellar ataxia is such a diagnostic pitfall. The clinical differentiation of OMS from acute cerebellar ataxia, the other major non-demyelinating neuroinflammatory ataxia in youngsters (26), is crucial both for the sake of the brain and the tumor. OMS rarely resolves without immunotherapy (acute cerebellar ataxia does) and it is paraneoplastic in half of the cases (acute cerebellar ataxia is not) (11). In the largest reported series of acute cerebellar ataxia in 73 children (12), onset age was older (not infants and toddlers), 81% had a viral prodrome, and 97% of cases were either varicella (26% of total) or illnesses presumed to be viral (52% of total). The non-exanthematous illnesses in OMS draw a sharp contrast.

In the present study, the time to OMS diagnosis of 1.2 months was shorter than the 2.8 months (range 0.01–32) found in our 2005 study (11), but still considered long in the context of active, early-onset, potentially reversible neuroinflammation (4). Although the level of evidence is low, two small case series suggest delayed diagnosis is related to more frequent long-term deficits. In an Italian case series of 14 patients (27), long-term deficits were more frequently detected in patients with an interval of more than 2 months between OMS onset and its diagnosis. In a Japanese case series (28), the median age at detection of a neuroblastic tumor in five patients with OMS was 7 months after OMS diagnosis, and the interval between OMS onset and detection and initial treatment of the neuroblastic tumor tended to be longer in patients with neurological sequellae. Larger outcome studies are needed. Making an early diagnosis in OMS has other merits, of course, such as sooner tumor detection, as well as improved quality of life afforded by earlier reduction of symptoms with treatment. The range of time to diagnosis of OMS, however, continues to be wide. In a Canadian case series (29), it was a median of 28 days (range 1–140), and in a US case series (30), it ranged from 2 days to 14 months. A campaign to boost OMS recognition should be nationwide, even international.

### Oncologic

Although it is often stated that 2–4% of neuroblastomas are associated with OMS (31), the data show that paraneoplastic frequency varies with patient age and is not a mere function of the frequency of neuroblastoma, owing perhaps to tumor or host factors, or both. Most notably, the frequency of OMS with neuroblastoma is the lowest during the first six months of life, when that of neuroblastoma without OMS is at its highest (31). The age at diagnosis for neuroblastoma without OMS is <1 years in 90%, 1–4 years in 68%, and 5–9 years in 52% (3). This novel disparity may be an important clue to the understanding of OMS pathophysiology. Possible host factors may include the developmental stage of the immune system (tolerance, surveillance, and antigen exposure) or of the brain (target antigens), and anti-tumor defenses (32, 33). Possible tumor factors may include tumor genomics and tumor immunological characteristics (34, 35). The now equal frequency of tumor and non-tumor designations may indicate better recent tumor detection. Extensive secondary analysis and inclusion of longitudinal data would be required to evaluate factors involved in long-term outcome, which is outside the scope of this report.

Another novel observation from the present study is the proportion of tumor types in OMS differs considerably from two other neuroblastic tumor-associated neurological paraneoplastic syndromes. In ROHHAD syndrome (rapid-onset obesity with hypothalamic dysfunction, hypoventilation, autonomic dysregulation), the breakdown of 23 cases with a tumor was 17% neuroblastoma, 22% ganglioneuroblastoma, and 61% ganglioneuroma (36). The tumor type proportions are the opposite of those in OMS, in which the rank order we found was 73% neuroblastoma, 22% ganglioneuroblastoma, and 4% ganglioneuroma. In pediatric anti-ANNA-1 (anti-Hu) paraneoplastic syndrome (limbic encephalitis/seizures), there have been no such tallies, but neuroblastoma (37, 38) and ganglioneuroblastoma (26, 39) have been reported, not ganglioneuroma to our knowledge. In an adult, anti-Hu antibodies and ganglioneuroblastoma were found, but with a different syndrome (cerebellar atrophy, progressive ataxia) (40). However, the sample sizes of ROHHAD and anti-Hu syndrome, the rarer disorders, are small, and the analysis cannot be more rigorous. The significance of these differences warrants further study.

Given that a paraneoplastic association was proven in only 50% of OMS cases and neuroblastoma may spontaneously regress (41) or be small enough to delay or evade detection, the designation of the remainder is uncertain (24, 30, 42). The requisite types of diagnostic scans and frequency of scanning are not standardized. Also, an argument for a para-/post-infectious designation has been made for specific infectious associations with OMS, such as Epstein–Barr virus (43), *Mycoplasma pneumoniae* (44), hepatitis C (45), adenovirus C3 (46), rotavirus (47), among others (4). In our study, however, mostly non-specific illnesses were reported, there was no consistent pathogen identified, and the majority of patients appeared to have no “infectious” prodrome. The clinical caution herein is that a seemingly viral illness does not preclude an underlying tumor (43). There is a need to better document whether infections evidence CNS/CSF involvement and to look for neuroblastic tumors in this patient population.

### Immunologic

A critical observation was that routine CSF tests, which showed little or no pleocytosis, missed the presence of neuroinflammation and immune cell dysregulation in OMS. They no longer comport with contemporary standards for diagnosing neuroinflammation. Also, the pattern of neuroinflammation in toddlers and youngsters was alike, indicating that developing brain is vulnerable at its most critical periods. Pathological expansion of CSF B cells and their intrathecal secretion of IgG OCB emphasize an important role for B cell and humoral immunity in OMS (22), not discounting T evidence of cell involvement (15). The pediatric OMS antigen/s, however, remain elusive despite the increasing comprehensiveness of commercial autoantibody panels, making the current panels not cost-effective for typical OMS. In contrast, CSF OCB analysis is available at more than one commercial laboratory, and fresh B cells can be measured by flow cytometry in the clinical laboratory at most hospitals once the protocol (15) and procedures (48) have been set up. The authors recommend testing for CSF OCB (even in the emergency department) at the initial diagnostic lumbar puncture in OMS. We have shown previously that the percentage of B cells in peripheral blood of children with OMS are not significantly elevated compared to controls (49). CSF continues to be the gold standard source for immunobiomarker measurements in neuroinflammatory disorders (50).

### Shifted Practice Patterns

This study demonstrated a shift in physician practice patterns toward the use of multiple immunotherapeutic agents for OMS. The reason for the shift can be questioned, but the answer would require a questionnaire study for treating physicians, and is otherwise speculative. One possibility is the yield of immunotherapeutics research, so clinical practice now includes a broader selection of treatment possibilities (30, 51–53) compared to our 2005 report (11), when no patients were on the anti-B-cell monoclonal antibody rituximab and few were on cyclophosphamide or other chemotherapeutic agents solely as anti-immune therapies. Another possibility is recent evidence that increased immunosuppression through delivery of multiple agents improves on developmental outcome of OMS (29, 30). In one case series (29), 8 of the 12 patients with OMS had been treated with multimodal immunotherapy (corticosteroids, IVIg, and an immunosuppressant agent, such as azathioprine, cyclophosphamide, rituximab)—10 patients had no or minimal neurological abnormalities. In another series (24), only 4 of 22 patients with OMS and a neuroblastic tumor received multimodal therapy—the rest corticotherapy—and 59% of the total patients had neurological sequellae. In a series of 14 patients (30), increased immunosuppression by multimodal therapy was associated with improved developmental outcome compared to previously reported children treated less intensively. In an observational study of 74 children with OMS (54), the multimodal therapy groups showed greater reduction in motor severity than single or dual agents. The current debate, which is between the front-end multimodal/multi-mechanistic therapy vs staggered or stepwise use of immunotherapeutic agents (4), is outside the scope of our study. Prospective studies are necessary for further evaluation of this issue (12).

### Study Strengths and Limitations

A notable strength was that all of the patients were evaluated by the same two experienced OMS investigators. Clinical severity was video-documented, and the videotapes allowed for blinded scoring of motor signs using a validated tool. Despite the rarity of OMS, a large and diverse group of children were enrolled, several-fold more than in previous studies, lending to sizable subgroups for analyses. There was broad representation across the US as to the demographic distribution, and racial and ethnic populations. The cohort was well characterized clinically, providing data for future secondary analyses and correlations for biomarker studies. Laboratory studies were standardized and centralized.

A limitation of the study was that more severe cases may have been referred to our OMS-specialty center, though we saw an admixture of severities. We may have drawn more patients closer to our center, but the center moved to three different regions and the patient draw was nationwide. Also, we saw more treated than untreated patients due to the logistics of getting acute cases to a single center before treatment, however, the number of untreated OMS was still large enough. The study confirms and updates laboratory early data we published on OCB (22), B cells (15), and limited autoantibodies (55), but the 3-fold increased sample size is quite substantial and the markers are commercially available to treating physicians. Marker results can vary significantly from lab to lab, but specific measures were taken to delimit variability. Inclusion of international patients with US patients may have added heterogeneity, though accounting for only 8% of the whole, and separate analysis international group showed mostly similarities.

## Conclusion

As acute cerebellar ataxia is a nearly universal initial misdiagnosis, the index of clinical suspicion for OMS should be escalated in the toddler with gait ataxia and irritability, even before the appearance of opsoclonus or myoclonus. Prodromal symptoms, regardless if suggestive of a specific illness, do not rule out neuroblastoma. The age-of-onset mismatch in frequency of neuroblastoma without OMS and neuroblastoma with OMS is a novel observation that may provide an important clue to OMS and neuroblastoma biology, involving host and tumor factors. Immunotherapy should be informed by the presence of positive CSF IgG OCB and B cells, which suggest an important role for B cell and humoral immunity in OMS (18), and not dissuaded by the usual absence of pleocytosis. Our findings highlight the need for increased recognition of OMS as an urgent and treatable condition.

## Ethics Statement

This study was carried out in accordance with the recommendations of the respective IRBs (CUMC IRB, Children’s National IRB, and SCRIHS) with written informed consent from the parents (subjects were below age of consent). The parents of all subjects gave written consent in accordance with the Declaration of Helsinki. The protocol was approved by CUMC IRB, Children’s National IRB, and SCRIHS. Additionally, Western IRB designated IRB exemption for retrospective analysis of demographic, clinical, and laboratory data.

## Author Contributions

MP conceptualized and designed the study, evaluated the patients, coordinated retrieval of the data, oversaw the analyses, interpreted the data, and drafted the initial manuscript; ET shared in the study design, acquisition of clinical data, coordinated and supervised clinical evaluations, scored videotapes, and critically reviewed and revised the manuscript; NM shared in the design of the study analysis, populated the database, performed statistical analysis and graphics, and critically reviewed the manuscript; all authors gave final approval of the version to be published. All authors agree to be accountable for the content of the work.

## Conflict of Interest Statement

The authors declare that the research was conducted in the absence of any commercial or financial relationships that could be construed as a potential conflict of interest.
